# Correction: Improving the inhibitory effect of CXCR4 peptide antagonist in tumor metastasis with an acetylated PAMAM dendrimer

**DOI:** 10.1039/c9ra90039j

**Published:** 2019-05-29

**Authors:** Changliang Liu, Hongyang Duan, Zijian Zhao, Wenzhe Li, Lilusi Ma, Xiaocui Fang, Chen Wang, Yanlian Yang

**Affiliations:** CAS Key Laboratory of Standardization and Measurement for Nanotechnology, CAS Key Laboratory of Biological Effects of Nanomaterials and Nanosafety, CAS Center for Excellence in Nanoscience, National Center for Nanoscience and Technology Beijing 100190 P. R. China fangxc@nanoctr.cn wangch@nanoctr.cn yangyl@nanoctr.cn +86-10-82545561 +86-10-82545559; Academy for Advanced Interdisciplinary Studies, Peking University Beijing 100871 P. R. China; University of Chinese Academy of Sciences Beijing 100049 P. R. China

## Abstract

Correction for ‘Improving the inhibitory effect of CXCR4 peptide antagonist in tumor metastasis with an acetylated PAMAM dendrimer’ by Changliang Liu *et al.*, *RSC Adv.*, 2018, **8**, 39948–39956.

The authors regret that the term “CXCL12” was incorrectly displayed as “CXCR12” in Scheme 1 and Fig. 6(a)–(c) in the original article. The correct versions of Scheme 1 and Fig. 6(a)–(c) are presented below.

**Scheme 1:**
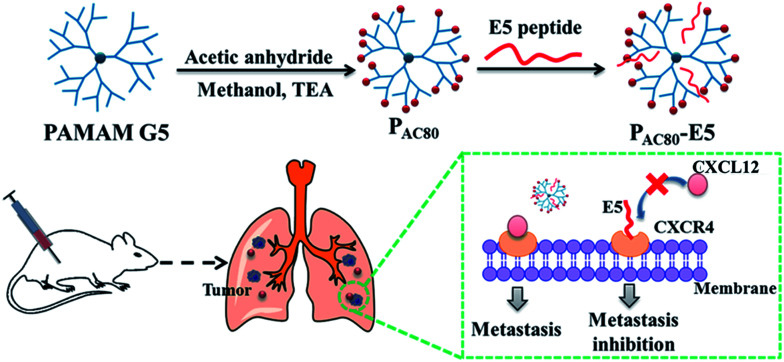
Schematic illustration of the preparation of the P_AC80_–E5 complex and the process of anti-tumor metastasis of the E5 peptide in the presence of P_AC80_.

**Fig. 6:**
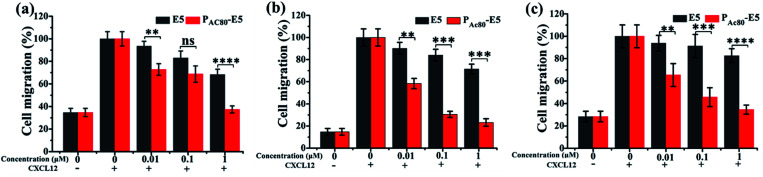
(a–c) The inhibitory effect of E5 and P_AC80_–E5 on: (a) MCF-7; (b) MDA-MB-231; and (c) 4T1 cells detected by transwell assay. The CXCL12 supplemented sample without E5 or P_AC80_–E5 was set as 100% as the control. Error bars represent the standard deviation (*n* = 3).

The Royal Society of Chemistry apologises for these errors and any consequent inconvenience to authors and readers.

## Supplementary Material

